# The Exclusive Title of Nursing Sister

**Published:** 1919-03-01

**Authors:** Geraldine Bremner

**Affiliations:** The Nurses' Co-operation, 22 Langham Street, W.1.


### THE EDITOR'S LETTER-BOX.

### The Exclusive Title of Nursing Sister.

*To the Editor of* THE HOSPITAL.

SIR,— In reference to your paragraph in THE HOSPITAL of February 22 advocating that the term "Nursing Sister" should be secured for all registered nurses, it may interest you to know that we adopted that term for the members of this staff two years ago. We realised that the time had come for this change.

GERALDINE BREMNER.

The Nurses' Co-operation,
22 Langham Street, W.1.

